# Identifying the narrative used by educators in articulating judgement of performance

**DOI:** 10.1007/s40037-019-0500-y

**Published:** 2019-03-26

**Authors:** Nyoli Valentine, Lambert Schuwirth

**Affiliations:** 1 ModMed, Adelaide, Australia; 2 Prideaux Centre, Flinders University, Adelaide, Australia

**Keywords:** Assessment, Narrative, Feedback

## Abstract

**Introduction:**

Modern assessment in medical education is increasingly reliant on human judgement, as it is clear that quantitative scales have limitations in fully assessing registrars’ development of competence and providing them with meaningful feedback to assist learning. For this, possession of an expert vocabulary is essential.

**Aim:**

This study aims to explore how medical education experts voice their subjective judgements about learners and to what extent they are using clear, information-rich terminology (high-level semantic qualifiers); and to gain a better understanding of the experts’ language used in these subjective judgements.

**Methods:**

Six experienced medical educators from urban and rural environments were purposefully selected. Each educator reviewed a registrar clinical case analysis in a think out loud manner. The transcribed data were analyzed, codes were identified and ordered into themes. Analysis continued until saturation was reached.

**Results:**

Five themes with subthemes emerged. The main themes were: (1) Demonstration of expertise; (2) Personal credibility; (3) Professional credibility; (4) Using a predefined structure and (5) Relevance.

**Discussion:**

Analogous to what experienced clinicians do in clinical reasoning, experienced medical educators verbalize their judgements using high-level semantic qualifiers. In this study, we were able to unpack these. Although there may be individual variability in the exact words used, clear themes emerged. These findings can be used to develop a helpful shared narrative for educators in observation-based assessment. The provision of a rich, detailed narrative will also assist in providing clarity to registrar feedback with areas of weakness clearly articulated to improve learning and remediation.

## What this paper adds

This paper sought to understand the characteristics of language that expert assessors use when supporting or clarifying their judgement of learners, with the intent of producing a shared narrative which can be used to support supervisors in translating their observations into feedback and decisions in real time.

## Introduction

Answers to the question ‘what is good assessment?’ have changed over the years. Previously, assessment was seen as a measurement problem and the literature was dominated by studies attempting to better understand factors influencing measurement properties of different assessment methods [1–3]. More recently, an increasing awareness and reappraisal of the role of human judgement in assessment has occurred. This was first seen when assessment of clinical competence moved back into the authentic context [4]. However, early implementations of assessments which incorporated human judgement, such as the mini-clinical evaluation exercise (mini-CEX) and objective structured clinical examinations (OSCE), were designed to minimize the influence of human judgement. Over time, it was noted that rater subjectivity was not the sole cause of unreliability (in fact, case specificity was actually far more important) [5] and it became apparent that expert human judgement is indispensable and not all factors can or should be controlled. Furthermore, researchers moved away from trying to identify a single best instrument to measure competence comprehensively and instead acknowledged that comprehensive assessment requires various instruments, including authentic assessment which requires human judgement, such as direct observation [6].

Human judgement plays a role in all forms of assessment. Even ‘objective’ multiple-choice examination involves a series of human judgements: the blueprint and its categories, choice of items, specific wordings, changes during the review process, and decisions about cut-off scores. There is, however, a fundamental difference between judgements in the design of a multiple-choice exam and judgements in observation-based exams. In the former, the human judgement processes are disconnected from the recording of candidate performance (the sitting of the examination), whereas in direct observation-based assessment the observation of candidate performance and judgement occur simultaneously in real-time. Therefore, direct observation-based assessments require the examiner to have sufficient expertise and experience in translating their observations to scores [7]. Kane describes this translation in the validity process as the inference from observation to score [8]. Many factors influence whether or not this translation occurs in a defensible and valid way [7].

Furthermore, human judgement in assessment is not undisputed. Often, human judgement is seen as too fallible to be used in high-stakes assessment. It has been said that human judgement is subjective and therefore unreliable. However, as already argued, every form of assessment involves human judgement. This is not a problem and some authors note a clear distinction between unreliability and subjectivity [9]. They argue that reliability or, in Kane’s validity theory, ‘universe generalization’, is a matter of inadequate sampling rather than of ‘objectivity’. Even ‘objective’ and standardized assessment with too few items will be unreliable. A large number of well sampled subjective judgements will lead to a reliable outcome. In a series of studies, Tverski and Kahnemann (summarized in [10]) demonstrate that human judgement is inferior to actuarial decisions.

However, it may be disputed whether assessment of medical competence is best represented by actuarial decision-making. Moreover, other fields of research, such as naturalistic decision-making, seek to understand why humans are still able to make ‘good enough’ decisions in information-poor situations and have a positive view on the role of biases and see them as elements of pattern recognition and quick decision-making [11, 12].

Human judgement in assessment appears acceptable provided there is a large number of observations, adequate sampling through all sources of variance and it is based on first-hand observations [1, 3]. However, these are precisely the challenges in workplace-based assessment: time is precious, assessment competes with healthcare duties, and unwanted sources of variance, such as case complexity and context, are nearly impossible to control.

An alternative approach to ensuring reliability and validity of scoring in assessment would be to ensure sufficient assessor ‘expertise’ [13–15]. Preliminary evidence suggests expertise impacts on the reliability and validity of workplace-based assessment. Weller et al. demonstrated that changing the wording of an assessment form from generic educational jargon to familiar clinical jargon had an effect on the number of observations needed to achieve acceptable generalizability coefficients for high-stakes assessment [16]. Instead of rating performance, supervisors judged the trainee’s level of independence, reaching a reliability coefficient of 0.70 with only nine assessments per trainee whereas the conventional assessment required 50 [16].

This study suggests that empowering assessors by giving them or allowing them to use language which fits their expertise has a positive impact on the psychometrics of the assessment. The way language supports and shapes human judgement and assessment has been further studied by Ginsburg et al. [17–20] through the exploration of the language consultants use in order to conceptualize trainees’ performance [18], identifying themes including knowledge, professionalism, patient interactions, team interactions, systems, disposition, trust and impact on staff. They suggest modern assessment is better served by embracing ‘subjectivity’ and idiosyncrasy of assessors, and there is valid information in the way the assessors describe their impressions.

A further study explored the interpretation of narrative comments in assessment and how this required ‘reading between the lines’ [19]. It suggests assessors use ‘code’ to avoid direct judgements and learners are able to ‘decode’. This may be because assessors feel ill-equipped for the task, and find it difficult to manage the uncertainty and ambiguity around observation-based assessment [21].

Govaerts et al. demonstrated many similarities in cognitive operations between clinical, diagnostic decision-making and making competence judgements about candidates [13, 14] suggesting the field of assessment can learn from clinical decision-making research. Bordage and Lemieux explored the role of semantic qualifiers in developing an understanding of decision-making ability or diagnostic expertise [22] and showed a relationship between diagnostic expertise and the command of higher level semantic qualifiers.

This leads to the question: what characteristics of language do expert assessors use when supporting or clarifying their judgement? Analogous to Bordage’s concept of semantic qualifiers one would expect that language assessment experts use would be information-rich and succinct [23]. Understanding this language is important for a better understanding of assessment expertise or ‘diagnostic decision-making’ in diagnosing ‘dyscompetence’.

The aim of our study was therefore to explore how medical education experts voice their subjective judgements about learners and to what extent they are using clear, information-rich terminology (high-level semantic qualifiers); and to gain a better understanding of the experts’ language used in these subjective judgements.

The intent of this understanding is not to produce a checklist but to understand a vocabulary. Creating and sharing a narrative vocabulary will help support assessors, substantiate their judgements in the complex task of translating observations to scores and giving meaningful formative feedback. Thus we seek to support both the summative and formative functions of observation-based assessment.

## Methods

This study was undertaken in general practice (GP) training in South Australia. Second year registrars (trainees) submitted six clinical case analyses which were structured ‘deconstructions’ of a clinical case. Registrars answered 18 questions describing the clinical presentation, differential diagnoses, investigations, legal and ethical issues, background medical knowledge and develop exam type questions. The case analyses are analyzed by medical educators (MEs).

### Participants and sampling

As we sought to understand the narrative used by assessors, we purposefully selected experienced assessors. Participants were MEs: practising GPs also working in education. There are ten MEs involved in GP training, and seven agreed to take part in the study. Of these, two MEs had 14 years of experience in education, one had 12 years of experience, two had 11 years of experience and one had 8 years of experience. Three were from rural locations and four from urban locations.

Each ME reviewed the most recent submitted case analysis in a ‘think out loud’ manner. They were instructed to verbalize their thoughts as they emerged. One author sat with the MEs and prompted them if there was ongoing silence and clarified any confusing statements. One-to-one interviews in a ‘think out loud’ manner was chosen to get as close to the original thinking of the educator as possible [24].

The sessions were recorded and transcribed verbatim by an independent transcriber. The time taken to review each case ranged from 26–57 min.

### Data analysis

Recorded sessions were analyzed using computer-assisted qualitative data analysis software (NVIVO version 10). A grounded theory approach was used as we wished for themes to emerge from the experience of the MEs. In the transcripts, initial codes were identified by one author to obtain as many narratives as possible from the data. These codes were then sorted and organized into focused themes by both authors. The data were rechecked to ensure no other themes had been overlooked. The data analysis continued until saturation was reached and no further codes were identified. Themes were ordered into higher order research themes by both authors in an iterative process until full agreement was reached.

Notes taken during the think out loud sessions were compared with the themes to ensure no relevant data were overlooked. In a member-checking process, the codes and themes were presented to participating MEs at a group meeting with an opportunity to clarify and add to the data.

Ethics approval was obtained from the Royal Australian College of General Practitioners Research and Evaluation Ethics Committee.

## Results

Consensus was frequently reached between the participating MEs. Saturation was reached after analysing five cases. Five themes emerged from the analysis. These were: (1) Demonstration of expertise; (2) Personal credibility; (3) Professional credibility; (4) Using a predefined structure and (5) Relevance. These are presented in Tab. 1.

**Table 1:** Themes emerging from the analysis

Theme	Subthemes
Demonstration of expertise	Completeness and Concision
	Depth
	Prioritization
	Purposefulness
	Plausibility
Personal credibility	Attention to detail
	Non-judgemental attitude/Empathy
	Demonstration of reflection/Commitment to lifelong learning
	Authenticity
Professional credibility	Using personal experience
	Articulating thought process
	Showing initiative/reliability
Using a predefined structure	–
Relevance	–

### Demonstration of expertise

The demonstration of well-organized knowledge and its purposeful application through presentation of a written case analysis was seen as a central issue for MEs. In the verbalization of what this actually meant, five subthemes emerged.

#### Completeness and concision

In demonstrating expertise, registrars need to find a balance between completeness and conciseness. That is, ensuring no relevant details are excluded but avoiding irrelevant details. To assess completeness, MEs sought evidence a patient had been comprehensively assessed and treated holistically. Completeness was therefore a judgement that sufficient relevant information had been elicited and used. It is a judgement analogous to the avoidance of construct underrepresentation in Messick’s validity theory [25].

*It’s short term, it’s long term, it’s preventions, it’s community, it’s the family. You have to touch on everything*

*I would have liked to have a little bit more information … about the nature of the bowel actions, was there blood in the bowel actions … mucous*

MEs looked for this to be balanced with concision, recognizing and summarizing important aspects of the patient’s presentation. Concision was judged on the basis of whether information was reported that was not sufficiently relevant to the problem—analogous to construct-irrelevant variance in validity [25].

*Succinct story at the start*

*So it gives you a bit of a background … doesn’t need to be much. It needs to have a little bit of detail but not too much*

The subthemes of depth, prioritization, purposefulness and plausibility helped inform the balance of completeness and concision.

#### Depth

Being thorough and recognizing the need to explore beyond the obvious presentation was seen as demonstration of expertise. Curiosity and depth of thinking was seen as being able to apply knowledge and skills to particular patient scenarios, that the registrar is ‘on top’ of the problem and has the cognitive reserve to dig deeper [26]. This involved being able to recognize one’s own knowledge deficits and learning needs. Depth was seen as a demonstration of expertise because put simply ‘what you don’t know you don’t notice’.

*So, is it actually … acute pain … he needed to exclude a whole lot of other things. For example, he needed to exclude any referred pain from a cardiac point of view, from a musculoskeletal point of view, from a pulmonary point of view*

*We don’t know if she’s had any vaginal discharge, any abnormal bleeding, still don’t know whether she’s compliant with her medication, so there’s … in my mind, a few holes in this*

#### Prioritization

MEs were looking for registrars to order items such as diagnoses or investigations according to relevance and clinical need. Prioritization can be seen as a level or organization of knowledge and better developed semantic networks [22, 27].

*When I look at investigations here, the thing that strikes me first is that some of these, you could argue, are more secondary tests rather than preliminary tests*

*I wouldn’t do thyroid second there. Probably third or fourth*

#### Purposefulness

Registrars were seen to demonstrate expertise if they could demonstrate their actions were driven by a hypothesis.

If the patient has got a bit of pallor you want to exclude bleeding

He has not really worked this up from the point of view of unconscious collapse

#### Plausibility

Putting forward a probable logical argument by means of correlating presenting symptoms with supporting history, exclusion of red flags, examination findings and investigations.

*That’s not what I’d be thinking … so I’d say to him: Can you explain to me why you’ve put that as your most likely single diagnosis?*

*And what is your diagnosis at this stage? Malignancy. I think that’s a bit of an interesting jump from a four-day acute onset of abdominal pain after being in Bali*

### Personal credibility

An assessor’s judgement on the registrar’s competency, especially in the domain of clinical reasoning and decision-making, is influenced by the registrar producing a *convincing* narrative/reasoning. Part of this is achieved through demonstration of attributes which are expected of professionals, such as trustworthiness, reliability, commitment to lifelong learning and attention to detail. Four subthemes emerged here.

#### Attention to detail

Personal credibility is influenced by attention to non-clinical details. The most obvious is grammar and spelling which could be argued should not matter, but MEs were susceptible to it. It was seen as an indication the registrar had taken the assignment seriously or as a sign of a well-organized mind.

*I just think it makes it look better if the English is spot on*

*Putting the right things in the right spots is important*

#### Non-judgemental attitude/Empathy

Registrars were seen to demonstrate personal credibility if they mindfully refrained from making judgements which were not clinically relevant. This may not be directly related to making the right diagnosis and/or therapeutic decisions, but was seen as an aspect of a well-developed professional identity. This also involved being able to view issues from the patient’s perspective.

*There’s no point in making value judgements but he has actually made some in that initial statement there*

*That’s really, really interesting: that he’s declined further investigation. And how do you sit with that? Do you sit comfortably or do you not?*

#### Demonstration of reflection/Commitment to lifelong learning

MEs assessed registrars on their ability to identify clues about their own strengths and weaknesses throughout the case and could translate these into actionable learning goals.

*What I’m looking for is that he’s reflecting mindfully on his knowledge gaps, … and found appropriate resources to improve on the gap. But the first bit is that I really want to see that he actually recognized what he’s missed on*

*So, the three main things you’ve learnt from this case: he’s actually written a little dissertation there on HIV seroconversion, chlamydia and he hasn’t done the reflections. He’s kind of put all of the relevant background medical science in there*

#### Authenticity

Registrars are required to use their own case and reference appropriately. Respecting intellectual ownership and generosity in admitting something is not one’s own work is seen to demonstrate personal credibility.

*This is all too much information, it doesn’t make sense for the registrar*

### Professional credibility

Credibility is further achieved when registrars demonstrate they have lived experience as a GP rather than just analytical knowledge. That is, the demonstration of understandings that cannot be learnt from books, but are acquired during practice. Three subthemes emerged to support professional credibility: using personal experience, articulating thought process and showing initiative/reliability.

#### Using personal experience

MEs were looking for registrars to be able to connect their personal experience with the case in a relevant and purposeful manner rather than relying on texts.

*… never heard of it. I’d ask her if she’s heard of it too or whether she just found it and looked it up*

#### Articulating thought process

Being able to articulate thought processes in a logical order through a clinical case gives the impression of being ‘on top’ of the problem and having reserve to reflect in action about their own decision-making.

*I’m thinking how did we get to SLE [systemic lupus erythematosus]?*

*So we have no idea what happened there*

#### Showing initiative/reliability

Being able to make and take responsibility for clinical decisions. Agency in ensuring every patient receives optimal treatment is not only seen as a demonstration of patient centredness but also of a mature professional attitude.

*Not just writing a referral letter and who knows if somebody sees it and deals with it in a proper time span*

### Using a predefined structure

Registrars are expected to have a structure to apply clinical information. This structure or framework may vary according to the context and registrar, but having a clear, coherent and plausible progression of ideas in a framework was valued by the MEs. They saw it as a demonstration of safety, minimizing the risk of important information and diagnoses being ignored.

*If they don’t ask structured questions, it makes it less likely they come to a diagnosis or at least exclude the red flags*

*Demonstrate a framework because the framework will keep them safe in general practice*

### Relevance

Relevance was seen as the ability to select a case and provide information relevant to being a GP. However, the data did not provide insight into what the MEs used to operationalize their conceptualization of relevance. The utterances remained largely at the level of gestalt or gut feeling.

*His references are cardiothoracic surgery … he could have done better from a GP point of view if he got a broader reference and looked at it from a broader [perspective]*

*I don’t care what intensivists do in HDU [high dependency units]*

## Discussion

This study found experienced MEs verbalize their judgements using high-level semantic qualifiers similar to how experienced clinicians verbalize their diagnostic judgements. The unpacking of these narratives allowed us to distil helpful themes. We do not presume these are the exact or only themes used by assessors; there most likely will be individual variability in the exact words used, despite the clear themes that emerged. In order to produce a universally shared narrative, replications in other contexts would be needed. Importantly these qualifiers were information-rich and yet succinct, making them useful for observation-based assessment.

We would argue against creating a checklist from the narrative or specific semantic qualifiers. Assessment of competence is not a matter of first ‘sharpening the pixels and then arbitrarily arranging them to see the whole picture’. Rather, it is seeing the whole picture, as a sort of top down processing, and then focusing on the individual pixels requiring further attention. To do this, assessment and content expertise are needed, as is a language to describe and manage what is observed during the assessment.

A clinical medicine analogy is that patients may present with different symptoms but yet have the same diagnosis. Symptoms are not used as a checklist to diagnose a condition, but rather clinicians accept diversity of different presentations.

The development of a vocabulary and possession of a shared narrative can facilitate a hand-over. Hand-overs are a continuity-of-care procedure which is essential in longitudinal assessment programs, in particular for programmatic assessment for learning. A shared narrative for this process is indispensable. Replication of our study in different contexts will be essential in determining shared narrative themes in evaluating learners and building a universal vocabulary.

Furthermore, a narrative helps make judgement more explicit and navigate uncertainty. Language plays a role in determining the fuzzy boundaries in complex situations. Through this, such a narrative will improve the ability of assessors to manage ambiguity and difficulties in providing judgement in observation-based assessment.

Finally, the possession of a clear and relevant narrative empowers and enables the assessor to provide the learner with constructive and concrete feedback, and the learner with a better ability to engage as a partner in the feedback for the learning process.

This is a small study and we do not expect our narratives to be precisely replicable in another context, but that was not our intent. We sought to explore two issues. Firstly, how medical education experts voice their judgements in clear, information-rich and succinct terminology. Secondly, how that terminology fits with a vocabulary or reference guide approach instead of being seen as a checklist of competence.
